# Search for common targets of lithium and valproic acid identifies novel epigenetic effects of lithium on the rat leptin receptor gene

**DOI:** 10.1038/tp.2015.90

**Published:** 2015-07-14

**Authors:** R S Lee, M Pirooznia, J Guintivano, M Ly, E R Ewald, K L Tamashiro, T D Gould, T H Moran, J B Potash

**Affiliations:** 1 Johns Hopkins Mood Disorders Center of the Department of Psychiatry and Behavioral Sciences, Johns Hopkins School of Medicine, Baltimore, MD, USA; 2 Graduate Program in Human Genetics, University of Maryland School of Medicine, Baltimore, MD, USA; 3 Departments of Psychiatry, Pharmacology, and Anatomy and Neurobiology, University of Maryland School of Medicine, Baltimore, MD, USA; 4 Department of Psychiatry, University of Iowa Carver College of Medicine, Iowa City, IA, USA

## Abstract

Epigenetics may have an important role in mood stabilizer action. Valproic acid (VPA) is a histone deacetylase inhibitor, and lithium (Li) may have downstream epigenetic actions. To identify genes commonly affected by both mood stabilizers and to assess potential epigenetic mechanisms that may be involved in their mechanism of action, we administered Li (*N*=12), VPA (*N*=12), and normal chow (*N*=12) to Brown Norway rats for 30 days. Genomic DNA and mRNA were extracted from the hippocampus. We used the mRNA to perform gene expression analysis on Affymetrix microarray chips, and for genes commonly regulated by both Li and VPA, we validated expression levels using quantitative real-time PCR. To identify potential mechanisms underlying expression changes, genomic DNA was bisulfite treated for pyrosequencing of key CpG island ‘shores' and promoter regions, and chromatin was prepared from both hippocampal tissue and a hippocampal-derived cell line to assess modifications of histones. For most genes, we found little evidence of DNA methylation changes in response to the medications. However, we detected histone H3 methylation and acetylation in the leptin receptor gene, *Lepr*, following treatment with both drugs. VPA-mediated effects on histones are well established, whereas the Li effects constitute a novel mechanism of transcriptional derepression for this drug. These data support several shared transcriptional targets of Li and VPA, and provide evidence suggesting leptin signaling as an epigenetic target of two mood stabilizers. Additional work could help clarify whether leptin signaling in the brain has a role in the therapeutic action of Li and VPA in bipolar disorder.

## Introduction

Bipolar disorder (BPD) is a debilitating mental illness that affects 3% of the adult population and constitutes a major global burden.^1^ It is a disease of extremes, characterized by mood states ranging from the highs of mania to the lows of depression. Despite intensive research, our grasp of BPD pathophysiology remains rudimentary. Pharmacological treatments are available, and while often effective, they fail to work in a substantial subset of patients. Their mechanism of action remains to be fully elucidated. Two of the most commonly prescribed BPD drugs are the mood stabilizers lithium (Li) and valproic acid (VPA; also formulated as divalproex sodium).

Studies that have sought to identify signal transduction pathways and genomic targets of these medications have begun to uncover epigenetic modifications that potentially underlie their mechanism of action. Previous studies have shown the role of Li in reducing inositol signaling and in subsequent regulation of neurotransmitter systems. Li has also been implicated in phosphorylation of AKT^2^ and inhibition of GSK-3 (glycogen synthase kinase 3),^3, 4^ both of which lead to the activation of the WNT pathway.

More recently, the role of Li in inositol and GSK-3β signaling has been shown to involve downregulation of DNA methyltransferase *Dnmt3a2* followed by reduction of DNA methylation (DNAm) at specific loci.^5^ Further, there is evidence that Li downregulates class I histone deacetylases (whose subtype includes HDAC1)^6^ and also causes locus-specific phosphorylation and acetylation of histone H3,^7^ suggesting a broader transcriptomic and epigenetic role for this drug. VPA, much similar to Li, can lead to phosphorylation of AKT and GSK-3β,^3, 8^ potentially affecting downstream signaling pathways, including the WNT pathway. In addition, VPA is a potent inhibitor of HDACs,^9^ which can influence DNAm.^10^

The effect of Li and VPA on AKT and WNT signaling pathways demonstrates the overlap in the targets of these dissimilar chemicals and provides our current rationale for seeking to identify and examine additional common targets. Given the differences in molecular structure—Li is a monovalent cation and VPA is a branched chain fatty acid—we hypothesized that molecular targets that are shared between the two drugs are more likely clinically relevant than are unique targets.^11, 12^ In these experiments, we sought to compare the mode of action of these mood stabilizers by studying their potential role in altering epigenetic patterns.

## Materials and methods

### Animals

Seven-week-old male Brown Norway rats (Charles River Laboratories, Frederick, MD, USA) were group-housed (three per cage) in a temperature- and humidity-controlled room under a 12  :12-h light:dark cycle. All animals received *ad libitum* access to water and standard laboratory chow (Harlan Teklad 2018, Frederick, MD, USA) for 1 week upon arrival, and experiments were initiated at 8 weeks of age. During the 4-week treatment period, animals were given a Li diet (*N*=12, 0.1% Li carbonate mixed into 2018 chow), a VPA diet (*N*=12, 0.2% sodium valproate mixed into 2018 chow) or the 2018 standard diet (*N*=12). Control animals were pair-fed to maintain non-distinguishable body weight differences for all animals. All animals were provided with 0.9% saline solution to prevent hyponatremia that may occur with Li treatment. Procedures were approved by the Institutional Animal Care and Use Committee at Johns Hopkins and performed in accordance with established guidelines.^13^

### Blood collection

Blood samples were collected weekly following onset of the light cycle (0900 hours) to measure plasma Li and VPA levels. Animals remained in a quiet room and ~250 μl of tail blood were collected from each.

### Tissue collection

Animals were euthanized by decapitation. Hippocampal tissues were dissected, frozen immediately on powdered dry ice and stored at −80 ^o^C.

### Expression microarray hybridization

Total RNA was obtained using the RNeasy Lipid Tissue Mini Kit (Qiagen, Germantown, MD, USA), and an aliquot of the total RNA samples extracted from the hippocampus were hybridized on the Affymetrix Rat Exon 1.0 ST microarrays (Santa Clara, CA, USA). Hybridization was performed for 16 h at 45 °C with constant rotation.

### Analysis of microarray data

Signal estimates and normalization for gene-level analysis were generated by a three-step analysis: background adjustment, quantile normalization and summarization.^14^ To reduce noise, probe sets and transcript clusters that fell into the lowest quartile of the expression signal distribution across all samples were excluded. Signal values were analyzed using the Bioconductor R package Robust Multichip Average algorithm.^14, 15^ Gene expression values were compared between the treatment groups using the moderated *t*-statistic of the Bioconductor package Limma.^16, 17^ To correct for multiple testing at the gene level, the Benjamini–Hochberg (FDR or false discovery rate) test was applied to identify differentially expressed genes (FDR-adjusted *P*-values <0.05). Significant up- and downregulated genes were subjected to functional enrichment analysis using DAVID.^18^ KEGG (Kyoto Encyclopedia of Genes and Genomes) analysis of genes associated with the list of probes from LI- or VPA-treated animals was performed to identify pathways that may be commonly affected by treatment. *Q*-values were determined by correcting the *P*-values obtained from the KEGG analysis by the total number of KEGG pathways.

### Quantitative PCR

QuantiTect Reverse Transcription Kit (Qiagen) was used on the remaining aliquot to generate complementary DNA for validation of microarray results by real-time quantitative PCR (qPCR). Negative reverse-transcribed samples were generated and all reactions were carried out in triplicate. Real-time reactions were performed on an Applied Biosystems 7900HT Fast Real-Time PCR System (Life Technologies, Grand Island, NY, USA). Each set of triplicates was checked to ensure that threshold cycle (Ct) values were within 0.25 Ct of each other. To determine relative expression values, the −ΔΔCt method was used, where triplicate Ct values for each sample were averaged and subtracted from those derived from housekeeping genes β-actin (*Actb*) and *Gapdh*. Similar results were obtained for normalization against both housekeeping genes.

### DNA extraction and bisulfite treatment

Genomic DNA from the hippocampus was isolated with the Masterpure DNA Purification Kit (Epicentre, Madison, WI, USA). An amount of 500 ng of the DNA was used for bisulfite conversion (EZ DNA Methylation-Gold Kit, Zymo Research, Irvine, CA, USA).

### Bisulfite PCR and pyrosequencing

We measured DNAm of specific regions within target genes by pyrosequencing of the PCR products.^19^ An amount of 25 ng of bisulfite-treated DNA was used for the initial PCR, and an additional nested PCR was performed with 2 μl of the previous PCR. Reactions were performed using the PyroMark MD System with Pyro Q-CpGt 1.0.9 software (Qiagen) for methylation quantification. Percentage of methylation at each CpG was compared between Li- and VPA- treated rats vs control diet-fed rats.

### Cell line

Rat hippocampal cell line H19-7/IGF-IR was obtained from ATCC (Manassas, VA, USA) and cultured in high-glucose DMEM media (Life Technologies) supplemented with 10% fetal bovine serum and 1x penicillin–streptomycin solution.

### Chromatin immunoprecipitation

Cultured cells were disaggregated by pipetting, and frozen rat brain tissues were finely chopped using a sharp scalpel. Cells and tissues were fixed using a 1% formaldehyde solution for 10 min on a rocking platform. The crosslinking procedure was quenched with 0.125 M glycine, and cells and tissues were washed three times with ice-cold PBS. Cultured cells and chopped tissues were resuspended in lysis buffer containing 1% Triton X-100 and further homogenized using a Dounce homogenizer to isolate the nuclei. Nuclei were subjected to centrifugation in a 30% sucrose gradient and ruptured with 1% SDS lysis buffer to release the chromatin. The chromatin solution was sonicated to yield ~250 bp DNA using the Diogenode Biodisuptor, and debris was cleared by centrifugation. An amount of 25 μg of chromatin was incubated with 10 μg of rabbit polyclonal antibodies against specific modifications on lysine residues of histone H3 and incubated for 2 h. Pre-immunization rabbit immunoglobulin G was used in generating the negative control chromatin samples, and an extra 25 μg of chromatin was used as input. Rabbit antibody-specific DynaBeads were used to precipitate and wash the chromatin complex. After five washes, proteins were digested by proteinase K, and eluates subjected to phenol–chloroform extraction followed by EtOH precipitation of DNA. Concentration of resuspended DNA was determined using a NanoDrop 1000 Spectrophotometer (Thermo Scientific, Wilmington, DE, USA).

### Chromatin immunoprecipitation quantitation

qPCR of specific regions within *Lepr* associated with various histone modifications was performed on the Applied Biosystems 7900HT Fast Real-Time PCR System as described above, except that SYBR green reagent was used for detection of PCR amplicons. Relative enrichment was calculated by comparing Ct values from 25 ng of chromatin immunoprecipitated DNA.

## Results

### Genome-wide expression and pathway analysis on changes in the hippocampus of rats treated with Li or VPA

**Figure 1:**
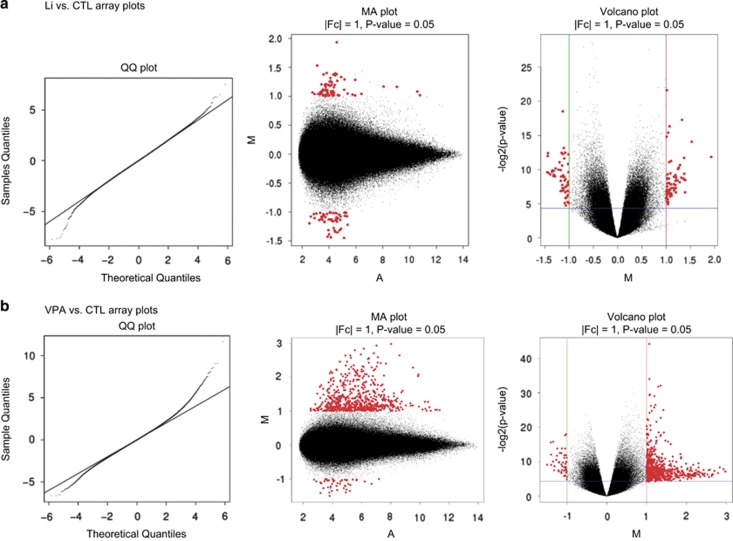
Lithium (Li) and valproic acid (VPA) exhibit distinct probe intensities for quantile–quantile (QQ), MA, and volcano plots. Observed intensities of probes for VPA vs control (CTL) show greater deviation from normal distribution than in Li vs CTL comparison (**a**, **b** left panels). MA plots depict expression quantity M (log_2_ [FC]) vs mean average (A) of probe intensities for Li and VPA, where the larger dots are those that satisfy absolute FC>2 (or M>1) and *P*-value <0.05 (**a**, **b** center panels). The volcano plots show expression quantity M (log_2_ [FC]) vs statistical significance (log_2_ [*P*-value]), where the larger dots are those that satisfy absolute FC>2 (or M>1) and *P*-value <0.05 (**a**, **b** right panels). For both the MA and volcano plots, VPA vs CTL comparison shows significantly more upregulated probes than downregulated probes. FC, fold change.

Three groups of Brown Norway rats received Li, VPA, or control (CTL) chow for 30 days. Levels of Li and VPA were 1.1±0.05 mM and 53.8±6.3 μg ml^−1^ at week 2 (mean±s.e.m.), and 1.0±0.07 mM and 37.9±5.5 μg ml^−1^ at week 4, respectively. These levels are within the human therapeutic range of Li or VPA,^20, 21^ and the treatment duration has been shown to attenuate stimulant-induced hyperlocomotion for Li^22^ and hyperactivity for VPA.^23^ Hippocampal mRNA was processed for hybridization on the Affymetrix microarray. We generated a normal quantile–quantile plot to compare the observed distribution of probe intensities against those of a normal distribution for Li and VPA (Figures 1a and b, left panels). The quantile–quantile plots show a greater deviation from the normal distribution for the VPA vs CTL comparison than for Li vs CTL, with probes upregulated by VPA being overrepresented. Further, MA plots that compare expression quantity M (log_2_ (fold change, FC)) vs mean average (A) of probe intensities for Li and VPA (Figures 1a and b, center panels) confirmed the high number of probes upregulated by VPA. Finally, a volcano plot depicting statistical significance (−log_2_[*P*-value]) vs M for Li and VPA (Figures 1a and b, right panels) compared with control diet-fed rats revealed dozens of probes up- or downregulated by both medications when the threshold was set at *P*<0.05 and |M|⩾1 (or absolute |FC|>2.0). Once again, we observed a disproportionately large number of probes upregulated with VPA, consistent with previous studies documenting VPA's role as a histone deacetylase inhibitor.^9^ In contrast, there were relatively few genes downregulated by VPA and relatively few genes whose expression was affected by Li. Top six most significant probe IDs and the associated genes affected by Li or VPA are included in Table 1, and the entire list is included in Supplementary Table 1.

We performed a KEGG analysis^24, 25^ to identify biologically interesting and potentially relevant pathways regulated by Li and VPA (Supplementary Table 2). We found 13 pathways that were associated with genes upregulated by VPA, including those involved in cell adhesion molecules and autoimmune thyroid disease, and one pathway, that is, carbohydrate digestion and absorption, associated with genes downregulated by Li. There were no pathways statistically significant by *Q*-value that were common between Li- and VPA-regulated genes. Further, no *Q*-value significant pathways were identified for genes downregulated by VPA or upregulated by Li. We also combined and analyzed the up- and downregulated genes for each group and observed no disease-relevant pathways in addition to the ones already observed for the previous analysis (Supplementary Table 2).

### Validation of common targets of Li and VPA by real-time qPCR

We then sought to identify array probes of genes commonly up- or downregulated by both medications. Of those with *P*⩽0.05 (treated vs controls), we identified 49 commonly upregulated array probes mapping to 36 genes and four downregulated probes mapping to four genes (Supplementary Table 3). Among these, we attempted to validate several commonly regulated genes with significant FC (⩾1.6 or ⩽0.8) difference between treated and control animals. We performed qPCR on *Akt1*, *Bace2*, *Dnah11*, *Glra1*, *Gulp1*, *Kcnj13*, *Lepr*, *Mmp2*, *Ogn* and *Slc6a20* using hippocampal mRNA. We validated a significant fold increase for most of these in response to both medications, exceptions being *Bace2, Glra1* and *Slc6a20*, where an increase for Li-treated samples was nonsignificant by *t*-test, and a predicted increase of *Slc6a20* in VPA-treated samples was not observed. *Akt1*, encoding protein kinase B, was the only gene downregulated by both treatments in the array data, and we observed a 20% decrease in this gene (0.8 FC) for both Li- and VPA-treated animals by qPCR. These results are shown in Table 2, and results for *Akt1* are consistent with previous literature, with most studies observing an increase in phosphorylation of the AKT protein, but either no change or a slight decrease in protein or mRNA expression levels with acute exposure to Li and VPA.^2, 26^ No studies have previously examined the effect of chronic exposure (~4 weeks) to mood stabilizers on *AKT1* expression.

### Lack of DNAm changes in Li- and VPA-treated hippocampal tissues

**Figure 2:**
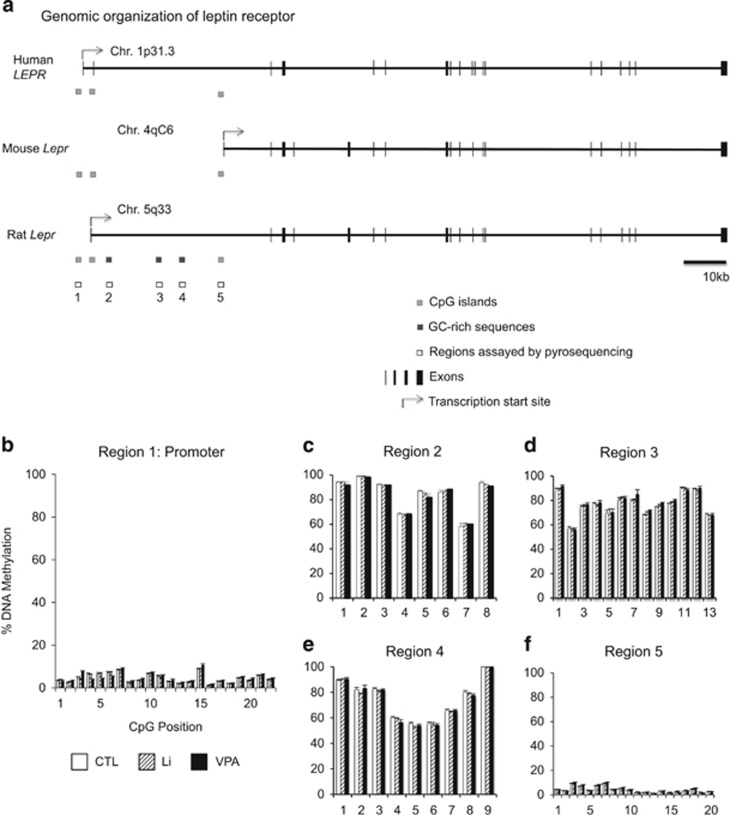
Lithium (Li) and valproic acid (VPA) treatments do not affect DNA methylation in the leptin receptor gene. Genomic organization of the leptin receptor gene in the human, mouse and rat shows three conserved CpG islands and three GC-rich sequences in the 5′ region (**a**). Consecutive CpG dinucleotides within two CpG islands and three GC-rich sequences were interrogated for determination of percent DNA methylation by bisulfite pyrosequencing (regions 1–5). First (5′) and last (3′) of the three CpG islands were hypomethylated, and corresponded to the rat promoter (**b**) and the homologous mouse promoter (**f**), respectively. The three intronic GC-rich sequences were hypermethylated (**c**–**e**). All data are presented as mean±s.e.m. None of the CpG comparisons between Li vs CTL or VPA vs CTL are statistically significant following correction for multiple testing.

Emerging evidence suggests that DNAm may have a role in the mechanism of action of Li and VPA. Of the genes commonly regulated by both medications, we focused on the leptin receptor gene (*Lepr*), as leptin signaling has been implicated in several studies of mood disorders and in the role of neurogenesis in these disorders.^27, 28, 29, 30, 31^ We asked whether upregulation of *Lepr* by chronic treatment with Li and VPA involved DNAm changes in the promoter and potential regulatory regions. We used bisulfite pyrosequencing to interrogate 72 CpGs in five regions. These regions included one conserved CpG island in the rat *Lepr* promoter, three GC-rich regions in the first intron and a conserved CpG island in the orthologous mouse promoter (Figure 2a). In all of these CpGs, we found little evidence of DNAm changes of substantial magnitude (that is, >10% Figures 2b–f). In addition, we also assayed the CpG islands and the surrounding ‘shore' regions of several of the commonly up- or downregulated genes in Table 2 and found little evidence of DNAm changes induced by these mood stabilizers. See Supplementary Table 4 for details.

### Histone modification of the leptin receptor gene in a cell line by Li and VPA

**Figure 3:**
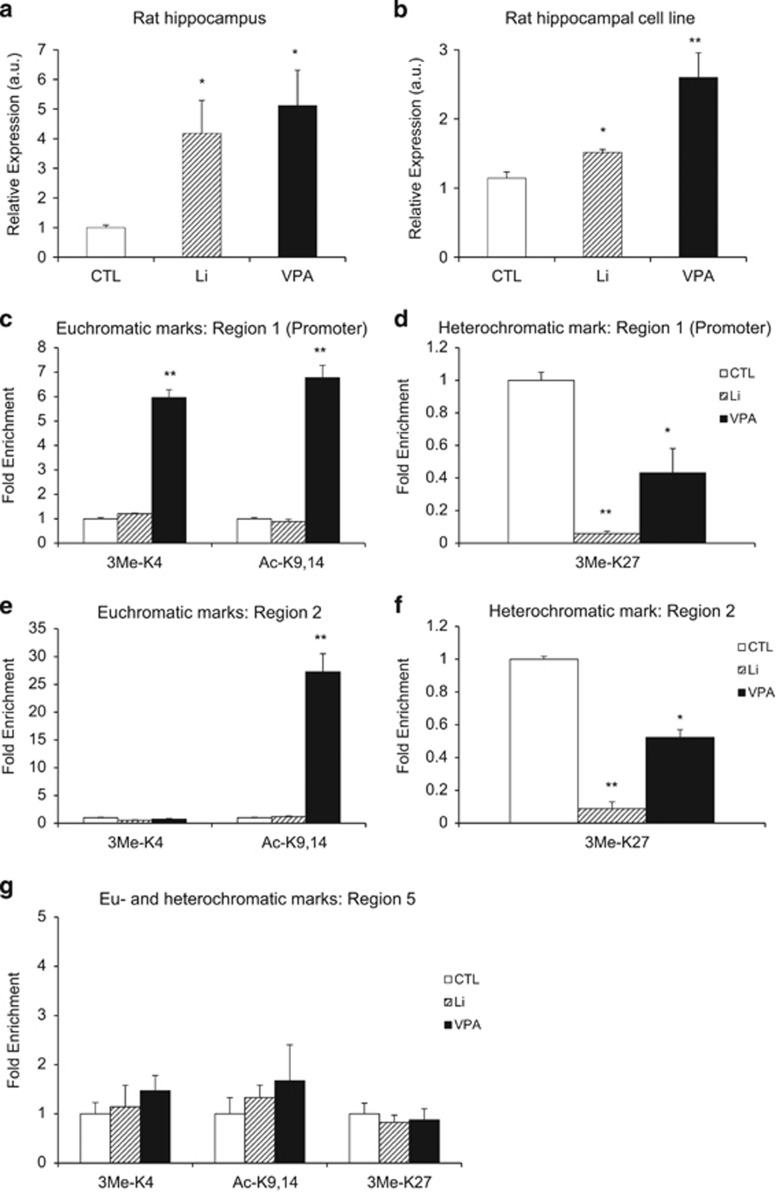
Treatment of the rat hippocampal cell line with lithium (Li) or valproic acid (VPA) led to changes in *Lepr* expression and histone modifications. Quantitative PCR using mRNA extracted from the rat hippocampal tissues (*N*=12 per group) was performed to validate the array results (**a**). Increase in expression of Lepr was also observed in the rat hippocampal cell line (*N*=3 per group) with 1 mM Li, 300 μM VPA or vehicle solution (**b**). Chromatin immunoprecipitation was performed to test three regions that corresponded to the rat promoter (**c**, **d**), intronic region (**e**, **f**) and the orthologous mouse promoter (**g**). Antibodies recognized trimethylated lysine-4 (3Me-K4), acetylated lysine-9,14 (Ac-K9,14) or trimethylated lysine-27 (3Me-K27) of histone H3. All data are presented as mean±s.e.m. *P<0.05 and **P<0.01. CTL, control.

We then used a cell system to dissect the mechanism involved, asking whether chronic exposure to Li and VPA could induce histone modifications in the *Lepr* promoter and potential regulatory regions (Figure 2a). As Li and VPA have been associated with histone deacetylase inhibition, we expected to observe an increase in association of the promoter with acetylated histones. We treated a rat hippocampal cell line with 1 mM Li or 300 μM VPA (~50 μg ml^−1^) for 5 days and collected genomic DNA, mRNA, and chromatin. Although 1-day treatment is sufficient to cause transcriptional changes,^2, 26^ we treated the cells for 5 days to effect epigenetic changes, which may take longer to establish. To ensure that transcription of *Lepr* in the cell line was similar to Li- and VPA-treated hippocampal tissues (Figure 3a), we first assessed for *Lepr* expression. We observed a 32.6% increase in *Lepr* for Li-treated cells (*P*=0.013) and a 127.4% increase for VPA-treated cells (*P*=0.008; Figure 3b). We next performed chromatin immunoprecipitation (ChIP) assays using antibodies against acetylated lysines-9,14 (Ac-K9,14-H3), trimethylated lysine-4 residue (3Me-K4-H3) and trimethylated lysine-27 residue (3Me-K27-H3) of histone H3. Both Ac-H3 and 3Me-K4-H3 are modifications associated with euchromatin, where the ‘open' histone conformation promotes gene transcription by increasing accessibility of its DNA to transcription factors. On the other hand, 3Me-K27-H3 is a modification associated with heterochromatin, or ‘closed' histone conformation, and promotes gene repression or silencing. Genes that lose trimethylation of K27-H3 can become transcriptionally active by ‘derepression.'^32^ We used the following antibodies: Ac-H3 for assessing VPA and Li action on histones; 3Me-K4-H3 for promoter activation; and 3Me-K27-H3 for measuring derepression of genes, such as those of the WNT pathway.^33^

qPCR against the *Lepr* promoter in ChIP samples revealed robust enrichment of the promoter with Ac-H3 and 3Me-K4-H3 in VPA-treated cells (Figure 3c). Association of the promoter with acetylated histone H3 supports previous work demonstrating VPA inhibition of histone deacetylase activity.^9^ In addition, these 3Me-K4-H3 ChIP results are consistent with association of lysine-4 methylation to gene promoters and with a previous study linking lysine-4 methylation with VPA.^34^ Although we observed no significant differences in enrichment of the promoter with the above two histone modifications in Li-treated samples, we did observe a marked reduction in representation of the region with 3Me-K27-H3 antibodies (Figure 3d). VPA treatment showed a similar, although lesser, reduction.

We also performed qPCR against a non-promoter, intronic region as a comparison. We observed a remarkably similar enrichment pattern, compared with the *Lepr* promoter, with Ac-H3 antibodies in VPA-treated samples (Figure 3e) and 3Me-K27-H3 antibodies in Li-treated samples (Figure 3f). Interestingly, we failed to observe an enrichment of the intronic region with 3Me-K4-H3 antibodies, supporting the role of this mark as promoter specific.^35, 36, 37^ Finally, we performed qPCR against the CpG island that corresponds to the orthologous mouse promoter region, as its high conservation (91.4%) to the mouse sequence may allow this region to act as a regulator of transcription in the rat. Nevertheless, it showed no significant differences in enrichment with the three histone modifications when Li- and VPA-treated samples were compared with the CTL group (Figure 3g). Differences in histone patterns at the rat promoter and the orthologous mouse promoter region are noteworthy, given the similar DNAm patterns observed (Figure 2).

### Validation of histone modifications in rat hippocampal tissues

**Figure 4:**
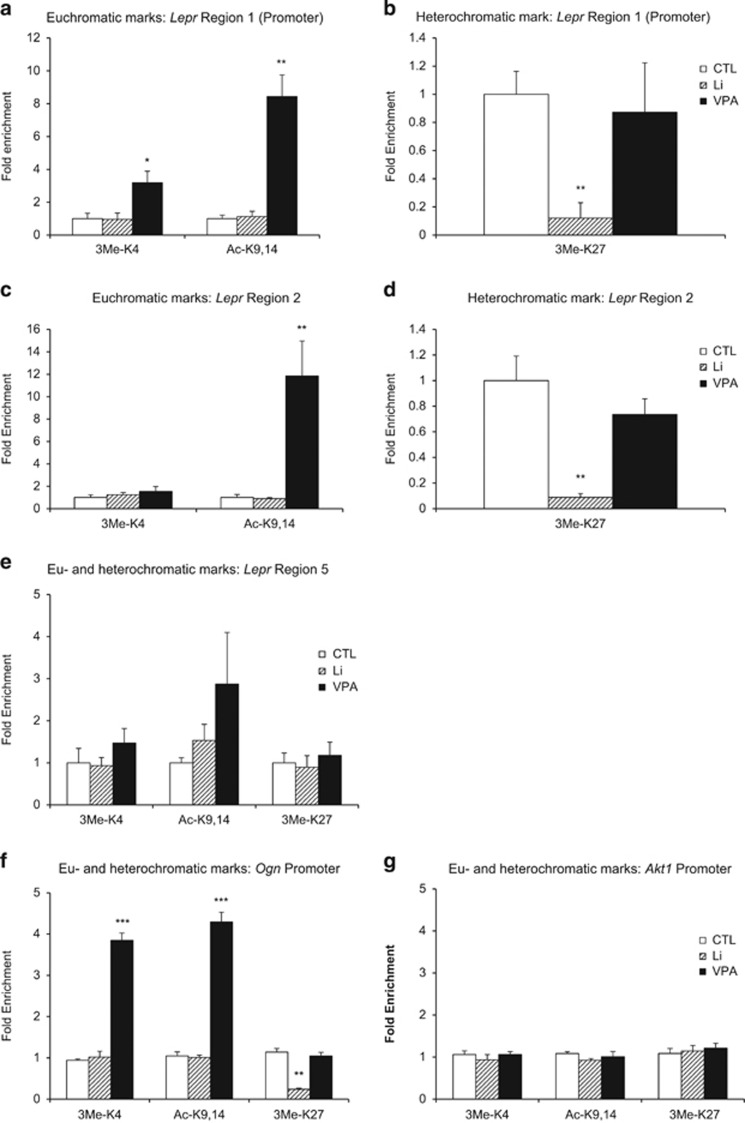
Treatment of rats for 30 days (*N*=4 per group) with lithium (Li) or valproic acid (VPA) led to changes in histone modifications at the *Lepr* gene. Chromatin immunoprecipitation was performed to test three regions that corresponded to the rat promoter (**a**, **b**), intronic region (**c**, **d**), the orthologous mouse promoter (**e**) and promoter regions of *Ogn* (**f**) and *Akt1* (**g**). Antibodies recognized trimethylated lysine-4 (3Me-K4), acetylated lysine-9,14 (Ac-K9,14) or trimethylated lysine-27 (3Me-K27) of histone H3. All data are presented as mean±s.e.m. *P<0.05, **P<0.01 and ***P<0.001. CTL, control.

Given the robust histone modifications in the cell line, we sought to replicate this finding in rat hippocampal tissues. To this end, we treated another cohort of rats with Li carbonate, VPA and control chow for 30 days, after which the rats were euthanized for tissue collection. Determination of drugs in the plasma resulted in similar values as in the first cohort (Li: 1.1±0.12 mM and VPA: 35.1±4.1 μg ml^−1^ at week 4). We isolated chromatin from the hippocampal tissues and performed the ChIP assay using the same antibodies as in the cell line. We found remarkably similar histone methylation patterns in the hippocampal tissues, with the exception that enrichment of the *Lepr* promoter with 3-Me-K4-H3 by VPA treatment was significantly lower in the hippocampus (Figure 4a), and that no significant reduction of both promoter and non-promoter regions was observed with 3-Me-K27-H3 antibodies in the VPA-treated samples (Figures 4b and d). In addition, ChIP–qPCR (Supplementary Table 5) of the orthologous mouse promoter region in the hippocampal tissues showed a similar pattern to that observed in the cell line (Figure 4e).

Finally, we examined the promoter regions of additional genes and found similar histone modifications in the commonly upregulated gene *Ogn* (Figure 4f), which has been implicated in neurite outgrowth,^38^ and no changes in the commonly downregulated gene *Akt1* (Figure 4g). Our results in the *Akt1* locus are consistent with its downregulation in Li- and VPA-treated animals being a secondary effect of AKT1 protein phosphorylation by Li and VPA rather than a direct drug response to Li or VPA.

## Discussion

Many genomic and pathway targets of Li and VPA have been identified over the course of the time they have been used as mood-stabilizing medications.^12, 39^ In addition to AKT1 and GSK-3, other notable targets include *Bcl-2*,^40^ *Gst*,^41^ *FEZ1*^42^ and *VEGFA,*^43^ which have been identified in diverse tissue types using different complementary DNA expression platforms. In particular, notable microarray studies using Li or VPA have identified a number of potential targets related to the stress response, the phosphatidylinositol cycle and synaptic transmission.^44, 45, 46, 47^ However, there has been very little overlap of target genes among these studies, presumably owing to differences in animal species, developmental period, brain region and treatment duration, among other reasons. Further, it is challenging to determine whether these targets are associated with therapeutic response or represent molecular effects that offer little benefit and cause unintended metabolic consequences.^11^ Although some of these studies have used microarrays to identify transcriptional actions of Li or VPA,^45, 46^ none, to our knowledge, have pursued a common transcriptomic approach combined with validation by qPCR and assessment of epigenetic regulation *in vivo*. To identify potential therapeutic targets, we hypothesized that there are common transcriptomic targets of Li and VPA in the rat hippocampus, with the presumption that those commonly regulated are more likely to be relevant for therapeutic response. Further, we focused on the hippocampus, as imaging,^48, 49, 50, 51, 52^ postmortem^53, 54, 55^ and pharmacological^52, 56, 57, 58^ evidence support its relevance for mood stabilizer studies.

We used the Affymetrix exon microarray platform and identified 40 genes commonly regulated by both drugs. Although many of the genes did not have known associations with BPD, Li or VPA, we selected and were able to validate by qPCR expression two genes that were previously shown to be involved in Li and VPA response (*Akt1* and *Mmp2*).^3, 8, 10^ We further validated genes with other known relationships to neuropsychiatric processes including processing of amyloid-β precursor proteins (*Bace2* and *Gulp1*),^59, 60, 61^ haloperidol exposure (*Glra1*),^62^ metabolism (*Lepr*) and epilepsy (*Kcnj13*).^63^ Of these genes, we were particularly interested in *Lepr* owing to its potential additional role in regulation of mood and anxiety. Besides having a role as a satiety hormone, leptin signaling has been implicated in depression and antidepressant response in humans,^29, 30^ and neurogenesis in an animal model of stress.^27^ In particular, *Lepr* deletion caused depression-like and anxiogenic behaviors and resistance to antidepressant-induced behaviors in animals.^28, 31, 64^ It remains to be seen to what extent leptin signaling in the brain may have a role in BPD. Only a few studies have been done on leptin signaling in BPD, and these have examined serum leptin levels primarily in the context of Li- or antipsychotic-induced weight gain.^65, 66, 67, 68, 69, 70^ Results have been mixed, with many reporting an increase in weight gain and plasma leptin levels with Li,^65^ VPA^71^ and antipsychotics,^66, 68^ and others reporting no significant change between BPD subjects and controls.^67, 69, 70^ To our knowledge, no human studies have examined how Li and VPA may affect leptin signaling in the brain.

To identify the underlying mechanism of gene regulation by chronic exposure to Li and VPA, we interrogated DNAm levels of several potential regulatory regions, such as the *Lepr* promoter, CpG island ‘shores' and three conserved GC-rich sequences. Although little change was observed in DNAm, there were significant changes in specific modifications of histone H3. VPA-mediated inhibition of HDACs and its effect on histone H3 acetylation is a well-established finding. On the other hand, reduction of the heterochromatic mark lysine-27 (K27) trimethylation of histone H3 constitutes a novel mechanism of transcriptional derepression for Li. It is unclear at this time whether both mood stabilizers can cause the observed loss of K27 trimethylation by inhibition of EZH2, the primary histone methyltransferase for H3K27 methylation. Intriguingly, EZH2 is phosphorylated and its activity is suppressed by AKT1.^32^ It is possible that the effect of Li on H3K27 methylation is mediated via AKT1 phosphorylation.^2^ In fact, a similar reduction, albeit to a lesser degree, of K27 trimethylation was observed with VPA treatment, which has also been shown to cause phosphorylation of AKT1. In addition, it is unknown whether *Lepr* or any of the other identified genes is a target of the WNT pathway. Further, we do not know how much of the observed epigenetic effects from Li and VPA are from binding of specific transcription factors to the *cis*-promoter elements. Although bioinformatic analyses have identified putative TCF3/LEF1 binding sites scattered across the *Lepr* locus, no *in vitro* or *in vivo* experiments have been performed. Additional work is necessary to clearly establish EZH2 as a potential target of these mood stabilizers and to implicate the WNT pathway in regulation of the common target genes.

The current study has several limitations. First, we chronically treated inbred, unperturbed rats with Li and VPA. Common targets were identified in the context of normal physiological conditions, which might have precluded those that compensate for a genetic lesion or restore molecular and behavioral deficits caused by adverse conditions, for example, stress exposure. As such, our data implicate an interesting gene in drug response, but do not directly demonstrate a role for leptin signaling in mood stabilization. In fact, similar epigenetic changes were observed with the gene that encodes osteoglycin (*Ogn*), a proteoglycan studied in the context of ectopic bone formation that has recently been implicated in neurite outgrowth.^38^ Although leptin signaling is particularly interesting in light of its association with anxiety and mood, it is also quite possible that a relatively less studied gene such as *Ogn* may be important for mood stabilization. Second, the *Lepr* expression increase observed in the drug-exposed animals could be due to metabolic consequences of the medications. However, hippocampal *Lepr* levels are not believed to be associated with metabolism, and the animals were pair-fed to minimize potential confounding factors associated with differential caloric intake. Further, our findings were replicated in the rat hippocampal cell line, an *in vitro* model completely devoid of the physiological context inherent in *in vivo* models. Third, while we did not observe DNAm changes in *Lepr*, these may exist elsewhere in the gene, as potential methylation-sensitive regulatory elements may be located outside of the candidate regions we examined. More comprehensive genome-wide studies are necessary to adequately address this.

Despite these limitations, our study demonstrates that Li and VPA regulate leptin receptor expression through epigenetic mechanisms. For Li, a pronounced reduction in H3K27 methylation was observed. The role of EZH2 in this process is unclear, as its levels were not measured in this study. Additional studies are needed to more carefully characterize this effect of Li and to determine whether leptin signaling in brain may have a role in BPD pathophysiology.

## Figures and Tables

**Table 1:** Genes affected by VPA or Li

*Gene*	*Probe set*	*Fold change*	P*-value*	Q*-value*
*Upregulated by VPA*				
*Slc16a10*	6260374	2.1	7.9 × 10^−12^	7.5 × 10^−6^
*Hmgcs2*	6171598	2.6	1.7 × 10^−11^	7.5 × 10^−6^
*Peci*	6498071	1.7	2.4 × 10^−10^	3.0 × 10^−5^
*Cox6b2*	5892608	2.1	3.2 × 10^−10^	3.0 × 10^−5^
*Ptgr1*	5974336	2.5	6.5 × 10^−9^	2.1 × 10^−4^
*Lrrc9*	6042599	2.0	1.1 × 10^−8^	2.9 × 10^−4^
				
*Upregulated by Li*				
*Hba-a2*	6227287	1.8	1.2 × 10^−6^	0.05
*H2-Ea*	5929473	1.7	6.2 × 10^−5^	0.30
*RGD1563741*	6192899	2.5	6.3 × 10^−5^	0.30
*Plg*	5919218	1.8	6.7 × 10^−5^	0.31
*LOC100134871*	5729215	1.8	8.1 × 10^−5^	0.34
*Cd93*	5784046	1.7	8.2 × 10^−5^	0.34
				
*Downregulated by VPA*				
*Plec1*	6654647	0.61	2.2 × 10^−5^	0.02
*LOC296111*	5971506	0.56	1.8 × 10^−4^	0.07
*Htra4*	6634189	0.48	2.6 × 10^−4^	0.09
*Vom2r72*	6005833	0.59	4.7 × 10^−4^	0.12
*RGD1560217*	6234656	0.56	5.2 × 10^−4^	0.13
*Mcemp1*	6674114	0.54	5.3 × 10^−4^	0.13
				
*Downregulated by Li*				
*Ebf2*	5711926	0.58	1.3 × 10^−4^	0.39
*RGD1562811*	5887714	0.59	2.6 × 10^−4^	0.49
*Klhl14*	6518251	0.47	5.2 × 10^−4^	0.57
*RGD1566226*	6210060	0.59	7.5 × 10^−4^	0.62
*Nsun2*	6531875	0.57	7.9 × 10^−4^	0.63
*Agmat*	5728878	0.57	0.001	0.67

Abbreviations: Li, lithium; VPA, valproic acid.

**Table 2:** Expression microarray prediction and qPCR validation

*Genes*	*Array fold change*		*Array* P*-value*		*qPCR fold change*		*qPCR* P*-value*	
	*Li*	*VPA*	*Li*	*VPA*	*Li*	*VPA*	*Li*	*VPA*
*Akt1*	0.6	0.6	0.002	0.004	0.8	0.8	**0.02**	**0.03**
*Bace2*	1.8	1.7	0.001	0.002	1.1	1.5	0.6	**0.02**
*Dnah11*	1.8	1.7	0.03	0.002	2.6	3.0	**0.03**	**0.002**
*Glra1*	1.7	1.6	0.007	0.03	1.3	1.7	0.3	**0.02**
*Gulp1*	1.7	2.4	0.005	4.4 × 10^−5^	1.3	2.1	**0.05**	**0.002**
*Kcnj13*	1.9	2.8	0.05	0.01	2.3	105	**0.03**	**0.002**
*Lepr*	1.7	2.0	0.01	6.0 × 10^−5^	4.2	5.1	**0.05**	**0.01**
*Mmp2*	1.6	2.3	0.004	3.5 × 10^−4^	1.4	3.1	**0.01**	**0.02**
*Ogn*	1.7	2.4	0.002	0.007	4.2	6.4	**0.003**	**0.04**
*Slc6a20*	1.6	1.7	0.001	0.005	1.8	1.0	0.2	0.8

Abbreviations: Li, lithium; qPCR, quantitative PCR; VPA, valproic acid. *P*-values ≤ 0.05 are in bold.

## References

[bib1] Merikangas KR, Jin R, He JP, Kessler RC, Lee S, Sampson NA et al. Prevalence and correlates of bipolar spectrum disorder in the world mental health survey initiative. Arch Gen Psychiatry 2011; 68: 241–251. 21383262 10.1001/archgenpsychiatry.2011.12 PMC3486639

[bib2] Chalecka-Franaszek E, Chuang DM. Lithium activates the serine/threonine kinase Akt-1 and suppresses glutamate-induced inhibition of Akt-1 activity in neurons. Proc Natl Acad Sci USA 1999; 96: 8745–8750. 10411946 10.1073/pnas.96.15.8745 PMC17587

[bib3] De Sarno P, Li X, Jope RS.. Regulation of Akt and glycogen synthase kinase-3 beta phosphorylation by sodium valproate and lithium. Neuropharmacology 2002; 43: 1158–1164. 12504922 10.1016/s0028-3908(02)00215-0

[bib4] Klein PS, Melton DA. A molecular mechanism for the effect of lithium on development. Proc Natl Acad Sci USA 1996; 93: 8455–8459. 8710892 10.1073/pnas.93.16.8455 PMC38692

[bib5] Popkie AP, Zeidner LC, Albrecht AM, D'Ippolito A, Eckardt S, Newsom DE et al. Phosphatidylinositol 3-kinase (PI3K) signaling via glycogen synthase kinase-3 (Gsk-3) regulates DNA methylation of imprinted loci. J Biol Chem 2010; 285: 41337–41347. 21047779 10.1074/jbc.M110.170704 PMC3009859

[bib6] Wu S, Zheng SD, Huang HL, Yan LC, Yin XF, Xu HN et al. Lithium down-regulates histone deacetylase 1 (HDAC1) and induces degradation of mutant huntingtin. J Biol Chem 2013; 288: 35500–35510. 24165128 10.1074/jbc.M113.479865 PMC3853296

[bib7] Kwon B, Houpt TA.. Phospho-acetylation of histone H3 in the amygdala after acute lithium chloride. Brain Res 2010; 1333: 36–47. 20346924 10.1016/j.brainres.2010.03.068 PMC2871962

[bib8] Chen G, Huang LD, Jiang YM, Manji HK. The mood-stabilizing agent valproate inhibits the activity of glycogen synthase kinase-3. J Neurochem 1999; 72: 1327–1330. 10037507 10.1046/j.1471-4159.2000.0721327.x

[bib9] Phiel CJ, Zhang F, Huang EY, Guenther MG, Lazar MA, Klein PS.. Histone deacetylase is a direct target of valproic acid, a potent anticonvulsant, mood stabilizer, and teratogen. J Biol Chem 2001; 276: 36734–36741. 11473107 10.1074/jbc.M101287200

[bib10] Milutinovic S, D'Alessio AC, Detich N, Szyf M. Valproate induces widespread epigenetic reprogramming which involves demethylation of specific genes. Carcinogenesis 2007; 28: 560–571. 17012225 10.1093/carcin/bgl167

[bib11] Gould TD, Quiroz JA, Singh J, Zarate CA, Manji HK. Emerging experimental therapeutics for bipolar disorder: insights from the molecular and cellular actions of current mood stabilizers. Mol Psychiatry 2004; 9: 734–755. 15136794 10.1038/sj.mp.4001518

[bib12] Gould TD, Chen G, Manji HK. Mood stabilizer psychopharmacology. Clin Neurosci Res 2002; 2: 193–212. 22707923 10.1016/S1566-2772(02)00044-0 PMC3375057

[bib13] National Research CouncilGuide for the Care and Use of Laboratory Animals. 8th edn, National Academies Press: Washington, DC, USA, 2011 xxv 220 p.

[bib14] Bolstad BM, Irizarry RA, Astrand M, Speed TP. A comparison of normalization methods for high density oligonucleotide array data based on variance and bias. Bioinformatics 2003; 19: 185–193. 12538238 10.1093/bioinformatics/19.2.185

[bib15] Irizarry RA, Bolstad BM, Collin F, Cope LM, Hobbs B, Speed TP. Summaries of Affymetrix GeneChip probe level data. Nucleic Acids Res 2003; 31: e15. 12582260 10.1093/nar/gng015 PMC150247

[bib16] Smyth GK. Linear models and empirical bayes methods for assessing differential expression in microarray experiments. Stat Appl Genet Mol Biol 2004; 3, Article3. 10.2202/1544-6115.1027 16646809

[bib17] Wettenhall JM, Simpson KM, Satterley K, Smyth GK. affylmGUI: a graphical user interface for linear modeling of single channel microarray data. Bioinformatics 2006; 22: 897–899. 16455752 10.1093/bioinformatics/btl025

[bib18] Huang, da W, Sherman BT, Tan Q, Kir J, Liu D, Bryant D et al. DAVID Bioinformatics Resources: expanded annotation database and novel algorithms to better extract biology from large gene lists. Nucleic Acids Res 2007; 35: W169–W175. 17576678 10.1093/nar/gkm415 PMC1933169

[bib19] Colella S, Shen L, Baggerly KA, Issa JP, Krahe R. Sensitive and quantitative universal Pyrosequencing methylation analysis of CpG sites. Biotechniques 2003; 35: 146–150. 12866414 10.2144/03351md01

[bib20] Gelenberg AJ, Kane JM, Keller MB, Lavori P, Rosenbaum JF, Cole K et al. Comparison of standard and low serum levels of lithium for maintenance treatment of bipolar disorder. N Engl J Med 1989; 321: 1489–1493. 2811970 10.1056/NEJM198911303212201

[bib21] Goodman LS, Brunton LL, Chabner B, Knollmann BrC. Goodman & Gilman's Pharmacological Basis of Therapeutics. 12th edn, McGraw-Hill: New York, USA, 2011, p 2084.

[bib22] Gould TD, O'Donnell KC, Picchini AM, Manji HK. Strain differences in lithium attenuation of d-amphetamine-induced hyperlocomotion: a mouse model for the genetics of clinical response to lithium. Neuropsychopharmacology 2007; 32: 1321–1333. 17151598 10.1038/sj.npp.1301254

[bib23] van Enkhuizen J, Geyer MA, Kooistra K, Young JW. Chronic valproate attenuates some, but not all, facets of mania-like behaviour in mice. Int J Neuropsychopharmacol 2013; 16: 1021–1031. 23164454 10.1017/S1461145712001198 PMC3920978

[bib24] Nakao M, Bono H, Kawashima S, Kamiya T, Sato K, Goto S et al. Genome-scale gene expression analysis and pathway reconstruction in KEGG. Genome Inform Ser Workshop Genome Inform 1999; 10: 94–103. 11072346

[bib25] Ogata H, Goto S, Sato K, Fujibuchi W, Bono H, Kanehisa M. KEGG: Kyoto Encyclopedia of Genes and Genomes. Nucleic Acids Res 1999; 27: 29–34. 9847135 10.1093/nar/27.1.29 PMC148090

[bib26] Pan JQ, Lewis MC, Ketterman JK, Clore EL, Riley M, Richards KR et al. AKT kinase activity is required for lithium to modulate mood-related behaviors in mice. Neuropsychopharmacology 2011; 36: 1397–1411. 21389981 10.1038/npp.2011.24 PMC3096809

[bib27] Garza JC, Guo M, Zhang W, Lu XY. Leptin restores adult hippocampal neurogenesis in a chronic unpredictable stress model of depression and reverses glucocorticoid-induced inhibition of GSK-3beta/beta-catenin signaling. Mol Psychiatry 2012; 17: 790–808. 22182938 10.1038/mp.2011.161 PMC3368076

[bib28] Guo M, Huang TY, Garza JC, Chua SC, Lu XY. Selective deletion of leptin receptors in adult hippocampus induces depression-related behaviours. Int J Neuropsychopharmacol 2013; 16: 857–867. 22932068 10.1017/S1461145712000703 PMC3612133

[bib29] Kloiber S, Ripke S, Kohli MA, Reppermund S, Salyakina D, Uher R et al. Resistance to antidepressant treatment is associated with polymorphisms in the leptin gene, decreased leptin mRNA expression, and decreased leptin serum levels. Eur Neuropsychopharmacol 2013; 23: 653–662. 23026132 10.1016/j.euroneuro.2012.08.010 PMC4221661

[bib30] Lawson EA, Miller KK, Blum JI, Meenaghan E, Misra M, Eddy KT et al. Leptin levels are associated with decreased depressive symptoms in women across the weight spectrum, independent of body fat. Clin Endocrinol (Oxf) 2012; 76: 520–525. 21781144 10.1111/j.1365-2265.2011.04182.x PMC3296868

[bib31] Liu J, Perez SM, Zhang W, Lodge DJ, Lu XY. Selective deletion of the leptin receptor in dopamine neurons produces anxiogenic-like behavior and increases dopaminergic activity in amygdala. Mol Psychiatry 2011; 16: 1024–1038. 21483433 10.1038/mp.2011.36 PMC3432580

[bib32] Cha TL, Zhou BP, Xia W, Wu Y, Yang CC, Chen CT et al. Akt-mediated phosphorylation of EZH2 suppresses methylation of lysine 27 in histone H3. Science 2005; 310: 306–310. 16224021 10.1126/science.1118947

[bib33] Wang L, Jin Q, Lee JE, Su IH, Ge K. Histone H3K27 methyltransferase Ezh2 represses Wnt genes to facilitate adipogenesis. Proc Natl Acad Sci USA 2010; 107: 7317–7322. 20368440 10.1073/pnas.1000031107 PMC2867706

[bib34] Nightingale KP, Gendreizig S, White DABradbury C, Hollfelder F, Turner BM. Cross-talk between histone modifications in response to histone deacetylase inhibitors: MLL4 links histone H3 acetylation and histone H3K4 methylation. J Biol Chem 2007; 282: 4408–4416. 17166833 10.1074/jbc.M606773200

[bib35] Bernstein BE, Kamal M, Lindblad-Toh K, Bekiranov S, Bailey DK, Huebert DJ et al. Genomic maps and comparative analysis of histone modifications in human and mouse. Cell 2005; 120: 169–181. 15680324 10.1016/j.cell.2005.01.001

[bib36] Heintzman ND, Stuart RK, Hon G, Fu Y, Ching CW, Hawkins RD et al. Distinct and predictive chromatin signatures of transcriptional promoters and enhancers in the human genome. Nat Genet 2007; 39: 311–318. 17277777 10.1038/ng1966

[bib37] Kim TH, Barrera LO, Zheng M, Qu C, Singer MA, Richmond TA et al. A high-resolution map of active promoters in the human genome. Nature 2005; 436: 876–880. 15988478 10.1038/nature03877 PMC1895599

[bib38] Jeong EY, Kim S, Jung S, Kim G, Son H, Lee DH et al. Enhancement of IGF-2-induced neurite outgrowth by IGF-binding protein-2 and osteoglycin in SH-SY5Y human neuroblastoma cells. Neurosci Lett 2013; 548: 249–254. 23714241 10.1016/j.neulet.2013.05.038

[bib39] Can A, Schulze TG, Gould TD. Molecular actions and clinical pharmacogenetics of lithium therapy. Pharmacol Biochem Behav 2014; 123C: 3–16. 10.1016/j.pbb.2014.02.004 PMC4220538 24534415

[bib40] Chen G, Zeng WZ, Yuan PX, Huang LD, Jiang YM, Zhao ZH et al. The mood-stabilizing agents lithium and valproate robustly increase the levels of the neuroprotective protein bcl-2 in the CNS. J Neurochem 1999; 72: 879–882. 9930766 10.1046/j.1471-4159.1999.720879.x

[bib41] Wang JF, Shao L, Sun X, Young LT. Glutathione S-transferase is a novel target for mood stabilizing drugs in primary cultured neurons. J Neurochem 2004; 88: 1477–1484. 15009649 10.1046/j.1471-4159.2003.02276.x

[bib42] Yu Z, Ono C, Kim HB, Komatsu H, Tanabe Y, Sakae N et al. Four mood stabilizers commonly induce FEZ1 expression in human astrocytes. Bipolar Disord 2011; 13: 486–499. 22017218 10.1111/j.1399-5618.2011.00946.x

[bib43] Sugawara H, Iwamoto K, Bundo M, Ishiwata M, Ueda J, Kakiuchi C et al. Effect of mood stabilizers on gene expression in lymphoblastoid cells. J Neural Transm 2010; 117: 155–164. 19949822 10.1007/s00702-009-0340-8

[bib44] Youngs RM, Chu MS, Meloni EG, Naydenov A, Carlezon WA Jr., Konradi C. Lithium administration to preadolescent rats causes long-lasting increases in anxiety-like behavior and has molecular consequences. J Neurosci 2006; 26: 6031–6039. 16738246 10.1523/JNEUROSCI.0580-06.2006 PMC4205587

[bib45] Bosetti F, Bell JM, Manickam P. Microarray analysis of rat brain gene expression after chronic administration of sodium valproate. Brain Res Bull 2005; 65: 331–338. 15811599 10.1016/j.brainresbull.2005.01.004

[bib46] Bosetti F, Seemann R, Bell JM, Zahorchak R, Friedman E, Rapoport SI et al. Analysis of gene expression with cDNA microarrays in rat brain after 7 and 42 days of oral lithium administration. Brain Res Bull 2002; 57: 205–209. 11849827 10.1016/s0361-9230(01)00744-4

[bib47] McQuillin A, Rizig M, Gurling HM. A microarray gene expression study of the molecular pharmacology of lithium carbonate on mouse brain mRNA to understand the neurobiology of mood stabilization and treatment of bipolar affective disorder. Pharmacogenet Genomics 2007; 17: 605–617. 17622937 10.1097/FPC.0b013e328011b5b2

[bib48] Noga JT, Vladar K, Torrey EF. A volumetric magnetic resonance imaging study of monozygotic twins discordant for bipolar disorder. Psychiatry Res 2001; 106: 25–34. 11231097 10.1016/s0925-4927(00)00084-6

[bib49] Bertolino A, Frye M, Callicott JH, Mattay VS, Rakow R, Shelton-Repella J et al. Neuronal pathology in the hippocampal area of patients with bipolar disorder: a study with proton magnetic resonance spectroscopic imaging. Biol Psychiatry 2003; 53: 906–913. 12742678 10.1016/s0006-3223(02)01911-x

[bib50] Colla M, Schubert F, Bubner M, Heidenreich JO, Bajbouj M, Seifert F et al. Glutamate as a spectroscopic marker of hippocampal structural plasticity is elevated in long-term euthymic bipolar patients on chronic lithium therapy and correlates inversely with diurnal cortisol. Mol Psychiatry 2009; 14: 647. 10.1038/mp.2008.26 18347601

[bib51] Giakoumatos CI, Nanda P, Mathew IT, Tandon N, Shah J, Bishop JR et al. Effects of lithium on cortical thickness and hippocampal subfield volumes in psychotic bipolar disorder. J Psychiatr Res 2015; 61: 180–187. 25563516 10.1016/j.jpsychires.2014.12.008 PMC4859940

[bib52] Nugent AC, Carlson PJ, Bain EE, Eckelman W, Herscovitch P, Manji H et al. Mood stabilizer treatment increases serotonin type 1A receptor binding in bipolar depression. J Psychopharmacol 2013; 27: 894–902. 23926239 10.1177/0269881113499204 PMC3784836

[bib53] Ruzicka WB, Subburaju S, Benes FM. Circuit- and diagnosis-specific DNA methylation changes at gamma-aminobutyric acid-related genes in postmortem human hippocampus in schizophrenia and bipolar disorder. JAMA Psychiatry 2015; 72: 541–551. 25738424 10.1001/jamapsychiatry.2015.49 PMC5547581

[bib54] Torrey EF, Barci BM, Webster MJ, Bartko JJ, Meador-Woodruff JH, Knable MB. Neurochemical markers for schizophrenia, bipolar disorder, and major depression in postmortem brains. Biol Psychiatry 2005; 57: 252–260. 15691526 10.1016/j.biopsych.2004.10.019

[bib55] Knable MB, Barci BM, Webster MJ, Meador-Woodruff J, Torrey EF. Stanley Neuropathology C. Molecular abnormalities of the hippocampus in severe psychiatric illness: postmortem findings from the Stanley Neuropathology Consortium. Mol Psychiatry 2004; 9: 544. 10.1038/sj.mp.4001471 14708030

[bib56] Du J, Creson TK, Wu LJ, Ren M, Gray NA, Falke C et al. The role of hippocampal GluR1 and GluR2 receptors in manic-like behavior. J Neurosci 2008; 28: 68–79. 18171924 10.1523/JNEUROSCI.3080-07.2008 PMC2763546

[bib57] Walz JC, Frey BN, Andreazza AC, Cereser KM, Cacilhas AA, Valvassori SS et al. Effects of lithium and valproate on serum and hippocampal neurotrophin-3 levels in an animal model of mania. J Psychiatr Res 2008; 42: 416–421. 17512948 10.1016/j.jpsychires.2007.03.005

[bib58] Zhou R, Gray NA, Yuan P, Li X, Chen J, Chen G et al. The anti-apoptotic, glucocorticoid receptor cochaperone protein BAG-1 is a long-term target for the actions of mood stabilizers. J Neurosci 2005; 25: 4493–4502. 15872096 10.1523/JNEUROSCI.4530-04.2005 PMC6725025

[bib59] Ahmed RR, Holler CJ, Webb RL, Li F, Beckett TL, Murphy MP. BACE1 and BACE2 enzymatic activities in Alzheimer's disease. J Neurochem 2010; 112: 1045–1053. 19968762 10.1111/j.1471-4159.2009.06528.x PMC2819564

[bib60] Azkona G, Amador-Arjona A, Obradors-Tarrago C, Varea E, Arque G, Pinacho R et al. Characterization of a mouse model overexpressing beta-site APP-cleaving enzyme 2 reveals a new role for BACE2. Genes Brain Behav 2010; 9: 160–172. 19840121 10.1111/j.1601-183X.2009.00538.x

[bib61] Beyer AS, von Einem B, Schwanzar D, Keller IE, Hellrung A, Thal DR et al. Engulfment adapter PTB domain containing 1 interacts with and affects processing of the amyloid-beta precursor protein. Neurobiol Aging 2012; 33: 732–743. 20674096 10.1016/j.neurobiolaging.2010.06.006

[bib62] Santoro ML, Ota VK, Stilhano RS, Silva PN, Santos CM, Diana MC et al. Effect of antipsychotic drugs on gene expression in the prefrontal cortex and nucleus accumbens in the spontaneously hypertensive rat (SHR). Schizophr Res 2014; 157: 163–168. 24893910 10.1016/j.schres.2014.05.015

[bib63] Winden KD, Karsten SL, Bragin A, Kudo LC, Gehman L, Ruidera J et al. A systems level, functional genomics analysis of chronic epilepsy. PLoS One 2011; 6: e20763. 21695113 10.1371/journal.pone.0020763 PMC3114768

[bib64] Guo M, Lu XY. Leptin receptor deficiency confers resistance to behavioral effects of fluoxetine and desipramine via separable substrates. Transl Psychiatry 2014; 4: e486. 25463972 10.1038/tp.2014.126 PMC4270309

[bib65] Atmaca M, Kuloglu M, Tezcan E, Ustundag B. Weight gain and serum leptin levels in patients on lithium treatment. Neuropsychobiology 2002; 46: 67–69. 12378122 10.1159/000065414

[bib66] Gergerlioglu HS, Savas HA, Celik A, Savas E, Yumru M, Tarakcioglu M et al. Atypical antipsychotic usage-related higher serum leptin levels and disabled lipid profiles in euthymic bipolar patients. Neuropsychobiology 2006; 53: 108–112. 16557041 10.1159/000092219

[bib67] Himmerich H, Koethe D, Schuld A, Yassouridis A, Pollmacher T. Plasma levels of leptin and endogenous immune modulators during treatment with carbamazepine or lithium. Psychopharmacology (Berl) 2005; 179: 447–451. 15565432 10.1007/s00213-004-2038-9

[bib68] McIntyre RS, Mancini DA, Basile VS, Srinivasan J, Kennedy SH. Antipsychotic-induced weight gain: bipolar disorder and leptin. J Clin Psychopharmacol 2003; 23: 323–327. 12920406 10.1097/01.jcp.0000085403.08426.f4

[bib69] Soeiro-de-Souza MG, Gold PW, Brunoni AR, de Sousa RT, Zanetti MV, Carvalho AF et al. Lithium decreases plasma adiponectin levels in bipolar depression. Neurosci Lett 2014; 564: 111–114. 24525248 10.1016/j.neulet.2014.02.005

[bib70] Tsai SY, Chung KH, Wu JY, Kuo CJ, Lee HC, Huang SH. Inflammatory markers and their relationships with leptin and insulin from acute mania to full remission in bipolar disorder. J Affect Disord 2012; 136: 110–116. 21962564 10.1016/j.jad.2011.08.022

[bib71] Verrotti A, Basciani F, Morresi S, de Martino M, Morgese G, Chiarelli F. Serum leptin changes in epileptic patients who gain weight after therapy with valproic acid. Neurology 1999; 53: 230–232. 10408570 10.1212/wnl.53.1.230

