# Supplementary material for: Search for common targets of lithium and valproic acid identifies novel epigenetic effects of lithium on the rat leptin receptor gene
Source: Transl Psychiatry. 2015 Jul 14;5(7):e600–. doi: 10.1038/tp.2015.90 (PMC5068731; doi:10.1038/tp.2015.90)
Supplement: Supplementary Table 2 [file tp201590x2.doc]

| **KEGG** | **Genes in Category** | **Genes**  **Observed** | **Genes**  **Expected** | **Enrichment** | **P-value** | **Q-value** |
| --- | --- | --- | --- | --- | --- | --- |
| **Upregulated by valproic acid** | | | | | | |
| Bile Secretion | 74 | 11 | 0.68 | 16.07 | 9.2x10^-11^ | 3.3x10^-7^ |
| Cell adhesion molecules (CAMs) | 156 | 13 | 1.44 | 9.01 | 2.9x10^-9^ | 1.0x10^-5^ |
| Phagosome | 185 | 13 | 1.71 | 7.60 | 2.2x10^-8^ | 8.0x10^-5^ |
| Arachidonic acid metabolism | 74 | 8 | 0.68 | 11.69 | 4.4x10^-7^ | 0.0016 |
| Graft-versus-host disease | 60 | 7 | 0.56 | 12.61 | 1.4x10^-6^ | 0.0050 |
| Allograft rejection | 62 | 7 | 0.57 | 12.20 | 1.8x10^-6^ | 0.0062 |
| Type I diabetes mellitus | 69 | 7 | 0.64 | 10.97 | 3.6x10^-6^ | 0.0130 |
| Autoimmune thyroid disease | 72 | 7 | 0.67 | 10.51 | 4.8x10^-6^ | 0.0173 |
| Viral myocarditis | 100 | 8 | 0.93 | 8.65 | 4.5x10^-6^ | 0.0159 |
| Gastric acid secretion | 74 | 7 | 0.68 | 10.22 | 5.8x10^-6^ | 0.0208 |
| Protein digestion and absorption | 78 | 7 | 0.72 | 9.70 | 8.3x10^-6^ | 0.0296 |
| Endocytosis | 230 | 11 | 2.13 | 5.17 | 1.1x10^-5^ | 0.0407 |
| Leukocyte transendothelial migration | 114 | 8 | 1.05 | 7.59 | 1.2x10^-5^ | 0.0421 |
| **Downregulated by lithium** | | | | | | |
| Carbohydrate digestion and absorption | 37 | 4 | 0.04 | 108.02 | 5.6x10^-8^ | 0.0002 |

**Supplementary Table 2** Pathways regulated by Li or VPA

| **KEGG** | **Genes in Category** | **Genes**  **Observed** | **Genes**  **Expected** | **Enrichment** | **P-value** | **Q-value** |
| --- | --- | --- | --- | --- | --- | --- |
| **Up- and down-regulated by valproic acid** | | | | | | |
| Bile secretion | 74 | 11 | 0.77 | 14.22 | 3.4x10^-10^ | 1.2x10^-6^ |
| Cell adhesion molecules (CAMs) | 156 | 13 | 1.63 | 7.97 | 1.2x10^-8^ | 4.3x10^-5^ |
| Arachidonic acid metabolism | 74 | 9 | 0.77 | 11.64 | 8.4x10^-8^ | 0.0003 |
| Phagosome | 185 | 13 | 1.93 | 6.72 | 9.2x10^-8^ | 0.0003 |
| Protein digestion and absorption | 78 | 9 | 0.82 | 11.04 | 1.3x10^-7^ | 0.0005 |
| Graft-versus-host disease | 60 | 7 | 0.63 | 11.16 | 3.1x10^-6^ | 0.0111 |
| Allograft rejection | 62 | 7 | 0.65 | 10.80 | 3.9x10^-6^ | 0.0139 |
| Viral myocarditis | 100 | 8 | 1.05 | 7.65 | 1.1x10^-5^ | 0.0386 |
| Tight junction | 131 | 9 | 1.37 | 6.57 | 1.1x10^-5^ | 0.0378 |
| Autoimmune thyroid disease | 72 | 7 | 0.75 | 9.30 | 1.1x10^-5^ | 0.0382 |
| Type I diabetes mellitus | 69 | 7 | 0.72 | 9.71 | 8.0x10^-6^ | 0.0287 |
| Hepatitis C | 127 | 9 | 1.33 | 6.78 | 8.2x10^-6^ | 0.0294 |
| Gastric acid secretion | 74 | 7 | 0.77 | 9.05 | 1.3x10^-5^ | 0.0457 |
| **Up- and down-regulated by lithium** | | | | | | |
| African trypanosomiasis | 35 | 6 | 0.13 | 46.4 | 3.4x10^-9^ | 1.2x10^-5^ |
| Malaria | 56 | 6 | 0.21 | 29.0 | 6.4x10^-8^ | 0.0002 |
| Carbohydrate digestion and absorption | 37 | 4 | 0.14 | 29.3 | 1.1x10^-5^ | 0.0386 |
